# Editorial: The association between HLA genes and autoimmune liver diseases

**DOI:** 10.3389/fimmu.2023.1175342

**Published:** 2023-03-10

**Authors:** Yun Ma, Nanda Kerkar

**Affiliations:** ^1^ Institute of Liver Studies, King’s College Hospital, Department of Inflammation Biology, Faculty of Life Sciences and Medicine, King’s College London, London, United Kingdom; ^2^ Division of Pediatric Gastroenterology, Hepatology and Nutrition, Golisano Childrens Hospital at Strong, University of Rochester Medical Center, Rochester, NY, United States

**Keywords:** autoimmunity in the liver, HLA genes, diversity, pathogenesis, cellular immunity, immunogenicity, microbiome, epigenetics

This Research Topic aims to establish the links between HLA and non-HLA genes in autoimmune liver disease (AILD) in a diverse population that is inclusive of different ages, races and ethnicities. This is translational with original research articles as well as state of the art mini-reviews summarizing current knowledge. In this collection, we included four laboratory-based investigations from Asia and Turkey, located Western Asia and southeastern Europe, Southwestern and Far East Asia; and 6 mini-reviews from United States, United Kingdom/Europe, South America and Canada. In a large cohort of patients with primary biliary cholangitis (PBC) from China, Huang et al., reported that four disease predisposition HLA genes, DRB1*08:03, DRB1*07:01, DRB1*14:05, and DRB1*14:54 were present in high frequency and majority had already developed cirrhosis, further confirming the link between HLA genes and liver disease severity. In addition, they reported a link between HLA genes and Lactobacillus, indicating possible role of microbiota in disease progression. Mulinacci et al. have focused their work on studying the immunogenetics of PBC, both HLA associated and non-HLA associated in the pre-genome wide association studies (GWAS) and post GWAS eras. The role of environment in increasing the risk of developing PBC was elucidated by studies in North-east of England where there was a higher prevalence in urban areas with strong coal-mining heritage and in New York city clusters of PBC were found in patients living in zip codes that contained or were adjacent to toxic waste sites. They also discuss using Antigen specific immunotolerance – tolerogenic vaccines - as well as utilizing nanotechnology to develop tolerogenic immune modifying nanopeptides as a therapeutic strategy in PBC.

Using metabolomics, Yang et al., identified inosine as a metabolite with immunomodulatory effects and noted that it was highly altered between DRB1*04:05 positive and negative Chinese patients with AIH. Administration of inosine in a murine model of Con-A induced acute AIH demonstrated its protective effect by attenuating hepatocyte apoptosis, preventing oxidative stress and inhibiting the activation and glycolysis of CD4^pos^ T effector cells. This may have potential in the development of future therapy of acute AIH. Lapierre and Alvarez discussed how the main genetic association in type 1 and type 2 AIH is with HLA class II genes and the HLA DR locus, and that a lesser association with non-HLA genes exists. Studies that implicate single nucleotide polymorphisms (SNPs) in genes including TNF-α, Vitamin D, and AIRE in increasing susceptibility to AIH have been included and they have also highlighted the use of animal models to study the effect of a particular genetic background or specific genetic mutation in the development of AIH. We learned from the investigation by Yuksel et al. that HLA DRB*11 was most prominent in Turkish patients with AIH. They report that in children with AIH, immunosuppression could severely reduce the number of regulatory B cells (B regs, CD20^pos^CD24^pos^CD38^pos^), which actually tended to be at a higher frequency than in healthy controls. On the other hand, immunoregulatory cells such as naïve and activated T regs (CD4^pos^FOXP3^high^CD45RA^neg^), fluctuate over time. Tacrolimus was reported to be a B reg-sparing drug. Autoimmune B effectors played a complex role in the pathogenesis of AIH, one of the hallmarks of AIH is positivity for autoantibodies, ANA, SMA in type 1 AIH (AIH-1) and LKM1 in type 2 AIH (AIH-2). AIH-1 and AIH-2 have partially overlapping HLA gene profiling, HLA-DR3 for both subtypes of AIH, HLA-DR7 is unique for AIH-2. While most studies in AIH analyse susceptibility of HLA alleles according to AIH subtypes, Cancado et al. have given an interesting twist by exploring the relationship between genetic markers of susceptibility linked to HLA with individual serological markers of disease, using celiac disease as a prototype.

Although there is still no clear answer to the question whether HLA genes play a decisive role in disease development and severity, Ahuja et al. described an important role played by non-HLA genes in AIH patients, not previously reported from India. They reported association of HLA DRB1*03 gene and the increased frequency of GG genotype of cytotoxic T-Lymphocyte-associated protein 4 (CTLA-4) CT60 mutation in a cohort of North Indian patients who were anti-SLA positive and poor responders to immunosuppressive therapy. Their investigation has reiterated that both HLA and non-HLA genes play a role in the disease manifestation of AIH. In the review focused on pediatric autoimmune liver diseases, Mack reports data from the largest cohort of children with AIH and autoimmune sclerosing cholangitis (ASC) from Kings College Hospital that showed that the presence of HLA DRB1*03 conferred the highest risk of AIH and ASC compared to healthy controls. Data from other large cohorts in Germany and South America are included as well as information on HLA genotypes that are protective from AILD. She has suggested several research initiatives including using modern technologies like HLA3D to isolate MHC-restricted autoantigens in AILD as well as designing novel therapies using nanotechnology.

The role of HLA and microbiome in the development of autoimmune liver disease is summarized succinctly by Beretta-Piccoli et al. and colleagues. The fact that 80% of the blood supply to the liver is from the splanchnic circulation, lends credence to the fact that gut microbiome and the many factors that influence it, be it changes in gut permeability, dysbiosis, translocation of microbial products into the circulation allowing molecular mimicry to trigger autoimmune inflammatory responses or simply changes in dietary intake, ethnicity or geography may have a profound influence in the development of autoimmune disease. This is also a fertile area for manipulation and drug discovery. There is a difference between the genetic risk and observed risk of autoimmune liver disease and in a beautifully written minireview illustrated with several tables and figures, Czaja has summarized existing knowledge on how epigenetics could influence not only the development of autoimmune liver diseases but also outcomes by altering key processes like DNA methylation and miRNA (microRNA). This has the potential to be another exciting area of new drug development. In conclusion, we hope that this collection of translational offerings will be of interest to both the clinician and the basic scientist and allow one to get updated on the current status of HLA and non-HLA in relation with AILD, with focus mainly on AIH and PBC, particularly from areas where such data was not previously available.

## Author contributions

All authors listed have made a substantial, direct, and intellectual contribution to the work and approved it for publication.

